# EHMTI-0018. CGRP receptor antagonists attenuate pain behavior induced by cortical spreading depression in freely moving rats

**DOI:** 10.1186/1129-2377-15-S1-F6

**Published:** 2014-09-18

**Authors:** A Filiz, N Tepe, E Boran, E Sajedeh, E Lars, H Bolay

**Affiliations:** 1 Neurology Department, Gazi University School of Medicine, Ankara, Turkey; 2 Neurology Department, Lund University, Lund, Sweden; 3 Neurology Department Neuropsychiatry Center, Gazi University School of Medicine, Ankara, Turkey

## Introduction

Cortical spreading depression (CSD) is implicated in migraine. Pain behavior is important for experimental migraine studies.

## Aims

In this study, the effect of CSD on pain and anxiety behavior in freely moving rats was investigated. The effect of CGRP receptor antagonist (MK-8825) on CSD induced behavior, and c-fos activation pattern in certain brain structures were evaluated to understand pain mechanisms.

## Methods

Study was approved the Institutional Animal Care and Use Committee and care and handling of animals were in accord with National Institute of Health guidelines. CSD was performed by topical KCl in awake rat; basal pain thresholds to mechanical and cold allodynia were evaluated. Rats were given either saline or CGRP receptor antagonist 30 or 100 mg/kg. After CSD induction, spontaneous behavior, ultrasonic vocalization was recorded; anxiety tested by elevated plus maze and mechanical and cold allodynia were evaluated. C-fos immunohistochemistry was performed to brain sections.

## Results

MK-8825 reversed CSD induced freezing, grooming, head shake and wet dog shake behavior; increased mechanical and cold allodynia thresholds. MK-8825 reduced c-fos positive cell number in ipsilateral trigeminal nucleus caudalis (TNC) and thalamic reticular nucleus. We found no co-localization with c-Fos and CGRP, CLR or RAMP1.

## Conclusions

CGRP receptor antagonists dose dependently attenuated CSD induced pain response. Besides blocking central transmission at TNC, CGRP receptor antagonists may also exert their effect on thalamic reticular nucleus.

No conflict of interest.

